# Two novel species of *Papiliotrema* (*Rhynchogastremataceae*, *Tremellales*) isolated from flowers in northern Thailand

**DOI:** 10.3897/mycokeys.137.199289

**Published:** 2026-07-22

**Authors:** Chirayut Kathongthung, Pratthana Kodchasee, Saowaluck Singsalood, Chanokned Senwanna, Pannida Khunnamwong, Jaturong Kumla, Nakarin Suwannarach

**Affiliations:** 1 Center of Excellence in Microbial Diversity and Sustainable Utilization, Chiang Mai University, Chiang Mai 50200, Thailand Biodiversity Center Kasetsart University (BDCKU) Bangkok Thailand; 2 Department of Biology, Faculty of Science, Chiang Mai University, Chiang Mai 50200, Thailand Department of Microbiology, Faculty of Science, Kasetsart University Bangkok Thailand; 3 Office of Research Administration, Chiang Mai University, Chiang Mai 50200, Thailand Center of Excellence in Microbial Diversity and Sustainable Utilization, Chiang Mai University Chiang Mai Thailand; 4 Department of Microbiology, Faculty of Science, Kasetsart University, Bangkok 10900, Thailand Department of Biology, Faculty of Science, Chiang Mai University Chiang Mai Thailand; 5 Biodiversity Center Kasetsart University (BDCKU), Bangkok 10900, Thailand Office of Research Administration, Chiang Mai University Chiang Mai Thailand

**Keywords:** Anthophilous yeasts, multilocus phylogeny, polyphasic identification, taxonomy, tropical area

## Abstract

The genus *Papiliotrema (Rhynchogastremataceae)*, which includes basidiomycetous yeast species, has a worldwide distribution. In this study, six *Papiliotrema* strains were isolated from flowers of *Allamanda cathartica*, *Bidens pilosa*, *Cananga odorata*, *Oncidium* sp., and *Plumeria obtusa* collected in Chiang Mai Province, northern Thailand, in 2024. An integrative taxonomic approach combining multilocus phylogenetic analyses using sequences of the internal transcribed spacer (ITS), the D1/D2 domains of the nuclear ribosomal large subunit (D1/D2), the largest subunit of RNA polymerase II (*rpb1*), the second-largest subunit of RNA polymerase II (*rpb2*), and translation elongation factor 1-alpha (*tef1-α*) with detailed phenotypic characterization revealed that these strains represent two novel species, namely *Papiliotrema anthoicola***sp. nov**. (holotype, SDBR-CMU883^T^) and *P. floralis***sp. nov**. (holotype, SDBR-CMU888^T^). Phylogenetic analysis clearly demonstrated that these taxa are distinct from other *Papiliotrema* species. Full descriptions, illustrations, and a phylogenetic tree showing the positions of the two *Papiliotrema* species identified in this study are provided.

## Introduction

*Papiliotrema* is classified in the family *Rhynchogastremataceae*, order *Tremellales*, class *Tremellomycetes*, and phylum *Basidiomycota* (Sampaio et al. 2002). This genus was initially described by Sampaio et al. (2002) based on a polyphasic taxonomic approach, with *P. bandonii* designated as the type species. Over the past decade, the number of species assigned to the genus *Papiliotrema* has increased substantially through the taxonomic reclassification of species previously placed in the genera *Auriculibuller*, *Bullera*, and *Cryptococcus*, as well as through the discovery of additional novel species, largely driven by molecular phylogenetic analyses of the internal transcribed spacer (ITS) region and the D1/D2 domains of the large-subunit ribosomal DNA (D1/D2), integrated with morphological, biochemical, and physiological data (Kurtzman et al. 2011; Liu et al. 2015a, 2015b). Members of this genus share several phenotypic features, including asexual reproduction by polar budding, production of yellowish-white to brownish-yellow colonies, absence of sugar fermentation, presence of ubiquinone Q-10 as the major respiratory quinone, and inability to assimilate nitrate (Liu et al. 2015b; Gao et al. 2025; Kodchasee et al. 2026). However, the sexual morph (teleomorph) has been reported in only three species, *P. bandonii*, *P. fusca*, and *P. plantarum* (Sampaio et al. 2002; Into et al. 2018; Khunnamwong et al. 2018a; Gao et al. 2025). To date, the genus *Papiliotrema* comprises 38 recognized species (Gao et al. 2025; Sun et al. 2025; Kodchasee et al. 2026; MycoBank 2026).

Members of *Papiliotrema* have been isolated from diverse habitats, including soil (Crestani et al. 2009; Sun et al. 2025), freshwater (Pagani et al. 2016), termite guts (Handel et al. 2016), salterns (Shin et al. 2005), subglacial ice (de Garcia et al. 2012), the atmosphere (Takashima et al. 2003; Pohl et al. 2006), and various plant tissues, including leaves (Golubev et al. 2003; Sampaio et al. 2004; Loung et al. 2005; Surussawadee et al. 2014; Yurkov et al. 2015; Into et al. 2018; Khunnamwong et al. 2018a; Tan and Shivas 2023; Jiang et al. 2024; Gao et al. 2025), bark (Ferreira-Paim et al. 2014; Sylvester et al. 2015), and the hypanthium (Crous et al. 2021). Additionally, several *Papiliotrema* species have been reported from floral parts of plants, including *P. bandonii* from the inflorescences of *Cortaderia selloana* (Sampaio et al. 2002), *P. miconiae* from flowers of *Miconia* sp. (Pagani et al. 2016), *P. chiangmaiensis* from flowers of *Zamioculcas zamiifolia*, *P. pollinicola* from flowers of *Catharanthus roseus*, and *P. tectonae* from flowers of *Tectona grandis* (Kodchasee et al. 2026). Other *Papiliotrema* species have also been reported from flowers, including *P. aurea*, *P. flavescens*, *P. fusca*, *P. japonica*, *P. laurentii*, *P. pseudoalba*, *P. rajasthanensis*, and *P. terrestris*, which were isolated from wildflowers (Min et al. 2012, 2013a, 2013b; Hyun et al. 2014a, 2014b; Han et al. 2015a, 2015b; Kim et al. 2020); *P. flavescens* from tea flowers of *Camellia sinensis* var. *assamica* (Kanpiengjai et al. 2023) and flowers of *Pachystachys lutea* (Kodchasee et al. 2026); *P. laurentii* from flowers of *Qualea grandiflora* (Vieira et al. 2025); and *P. aspenensis* from flowers of *Plumeria pudica* (Kodchasee et al. 2026).

Yeasts associated with flowers, known as anthophilous yeasts, can influence floral scent composition, pollination dynamics, and the reproduction of their host plants. Anthophilous yeasts are found in nectar, petals, stamens, and pistils, which provide a nutrient-rich environment (Mittelbach et al. 2015; Canto et al. 2017; Klaps et al. 2020; Bogo et al. 2021; Jacquemyn et al. 2021; Kodchasee et al. 2026). Floral nectar contains high concentrations of sugars, whereas pollen is rich in proteins and amino acids that support yeast colonization (Pozo et al. 2011; Peay et al. 2012). Therefore, flowers are important habitats for yeast diversity. Thailand, located in the tropics and harboring 15,000 plant species (approximately 8% of the global total), provides diverse floral habitats and has been recognized as a hotspot for yeast biodiversity associated with tropical plants and flowers (Surussawadee et al. 2014; Into et al. 2018; Khunnamwong et al. 2018a; Donnelly 2024; Kodchasee et al. 2026). During an investigation of anthophilous yeasts associated with various flowers in northern Thailand in 2024, six yeast strains (SDBR-CMU883, SDBR-CMU888, SDBR-CMU892, SDBR-CMU893, SDBR-CMU894, and SDBR-CMU895) belonging to the genus *Papiliotrema* were isolated. This study aimed to identify these strains using an integrative polyphasic approach. The data obtained indicate that these strains represent two novel species. Their illustrations and detailed descriptions based on morphological characteristics, phenotypic analyses, and multilocus phylogenetic analyses are provided.

## Materials and methods

### Sample collection and yeast isolation

Healthy flower samples were collected in Chiang Mai, northern Thailand, in 2024. The samples were placed in sterile plastic bags, stored at 4 °C, and processed for yeast isolation within 24 h of collection. The internal parts of the flowers were aseptically suspended in 5 mL of 0.85% (w/v) saline solution for 15 min. The suspension was spread onto yeast extract-malt extract agar (YMA) plates containing 0.3% yeast extract, 0.3% malt extract, 0.5% peptone, 1.0% glucose, and 2.0% agar; supplemented with 50 mg/L chloramphenicol; and incubated at 25 °C for 3 days. Individual yeast colonies were selected and subcultured on YMA. The purified strains were preserved long term in 20% (v/v) glycerol at –80 °C and deposited at the Sustainable Development of Biological Resources Culture Collection (SDBR-CMU), Faculty of Science, Chiang Mai University, Chiang Mai, Thailand; the Thailand Bioresource Research Center (TBRC), Pathum Thani, Thailand; and the Guizhou Medical University Culture Collection (GMBCC), Guizhou, China. New yeast taxa were registered in the MycoBank database (MycoBank 2026).

### Morphological characterization

Morphological characteristics were examined following the standard methods described by Kurtzman et al. (2011) and Shibayama et al. (2020). Colony morphology was observed on YMA after incubation at 25 °C for 5 days. The formation of pseudohyphae and true hyphae was assessed through slide culture on potato dextrose agar (PDA) and cornmeal agar (CMA) incubated at 25 °C for 2 weeks. Basidiospore formation was investigated for individual strains on YMA, CMA, PDA, 5% malt extract agar (5% malt extract and 1.5% agar; 5% MEA), Fowell’s acetate agar (0.5% sodium acetate and 2% agar), and Gorodkowa agar (0.1% glucose, 0.5% sodium chloride, 1% peptone, and 2% agar) at 25 °C for 1 month. Micromorphological features were observed and photographed using a light microscope (Nikon Eclipse Ni-U, Tokyo, Japan). Capsule presence was determined by negative staining with India ink. Yeast cells and pseudohyphae were measured using the Tarosoft® Image Framework program, with at least 50 measurements obtained for each structure.

### Biochemical and physiological characterization

The biochemical and physiological characteristics of the novel yeasts were assessed following the standardized protocols described by Kurtzman et al. (2011). Carbon and nitrogen assimilation tests were conducted in liquid medium, and starved inocula were used for the nitrogen assimilation tests. Fermentation of glucose was examined in liquid medium using Durham fermentation tubes. Cycloheximide resistance was also evaluated in liquid medium. Acid production was investigated on solid media. Growth at various temperatures was determined on YMA at 10, 15, 25, 30, 37, and 45 °C. Tolerance to high osmotic pressure was assessed on media containing 50% or 60% glucose and on media containing 10% or 16% sodium chloride (NaCl) supplemented with 5% glucose.

### DNA extraction, PCR amplification, and sequencing

Genomic DNA was extracted from yeast cells grown in yeast extract-malt extract (YM) broth at 25 °C with shaking at 110 rpm for 48 h using a DNA Extraction Mini Kit (Omega Bio-tek, USA), following the manufacturer’s protocol. The internal transcribed spacer (ITS) region and D1/D2 domains of the large subunit ribosomal DNA (D1/D2) were amplified by polymerase chain reaction (PCR) using the primer pairs ITS1/ITS4 (White et al. 1990) and NL1/NL4 (Kurtzman and Robnett 1998), respectively. In addition, three protein-coding genes were amplified: the largest subunit of RNA polymerase II (*rpb1*), the second-largest subunit of RNA polymerase II (*rpb2*), and translation elongation factor 1-alpha (*tef1-α*), using the primer pairs RPB1-Af/RPB1-Cr, fRPB2-5F/fRPB2-7cR, and EF1-983F/EF1-2218R (Wang et al. 2014), respectively. The PCR mixture contained 7 µL of double-distilled water (ddH_2_O), 10 µL of 2 × Quick Taq™ HS DyeMix (TOYOBO, Japan), 1 µL of genomic DNA, and 1 µL of each forward and reverse primer. The PCR thermal cycling program for amplification of the ITS region and D1/D2 domains was as follows: an initial denaturation step at 95 °C for 5 min, followed by 35 cycles of denaturation at 94 °C for 30 s, annealing at 52 °C for 45 s, elongation at 72 °C for 1 min, and a final extension at 72 °C for 10 min. The PCR thermal cycling programs for amplification of *rpb1*, *rpb2*, and *tef1-α* were as follows: an initial denaturation step at 95 °C for 3 min, followed by 40 cycles of denaturation at 95 °C for 30 s, annealing at the specific temperature for each gene (56 °C for *rpb1*, 52 °C for *rpb2*, and 55 °C for *tef1-α*) for 45 s, elongation at 72 °C for 1 min, and a final extension at 72 °C for 10 min. PCR products were examined by electrophoresis on 1% agarose gels and then purified using a PrimeWay Gel Extraction/PCR Purification Kit (1^st^ Base Company, Malaysia). The purified PCR products were directly sequenced at the 1^st^ Base Company (Kembangan, Malaysia). All nucleotide sequences obtained in this study were deposited in GenBank (Table 1).

**Table 1.** Information on *Papiliotrema* species and strains used in the phylogenetic analyses and their hosts, localities, and GenBank accession numbers.

**Species**	**Strain**	**Host**	**Country**	**GenBank accession numbers**				
				**ITS**	**D1/D2**	***rpb1***	***rpb2***	***tef1-α***
*Papiliotrema anemochoreia*	CBS 10258^T^	Air sample	South Africa	KY104455	KY108727	KF036344	KF036758	KF037029
***Papiliotrema anthoicola***	**SDBR-CMU883^T^**	**Flowers of ylang-ylang (*Cananga odorata*)**	**Thailand**	**PZ298074**	**PZ298093**	**PZ309733**	**PZ309739**	**PZ309745**
***Papiliotrema anthoicola***	**SDBR-CMU893**	**Flowers of frangipani (*Plumeria obtusa*)**	**Thailand**	**PZ298075**	**PZ298094**	**PZ309734**	**PZ309740**	**PZ309746**
***Papiliotrema anthoicola***	**SDBR-CMU894**	**Flowers of ylang-ylang (*Cananga odorata*)**	**Thailand**	**PZ298076**	**PZ298095**	**PZ309735**	**PZ309741**	**PZ309747**
***Papiliotrema anthoicola***	**SDBR-CMU895**	**Flowers of golden trumpet (*Allamanda cathartica*)**	**Thailand**	**PZ298077**	**PZ298096**	**PZ309736**	**PZ309742**	**PZ309748**
*Papiliotrema aspenensis*	CBS 13867^T^	Bark of a trembling aspen (*Populus tremuloides*)	USA	NR158801	NG060109	–		–
*Papiliotrema aspenensis*	SDBR-CMU652	Flowers of bridal bouquet (*Plumeria pudica*)	Thailand	PV834666	PV834496	–	–	–
*Papiliotrema aurea*	CBS 318^T^	The atmosphere	Japan	NR130650	NG148937	LK024017	KF036763	LK023956
*Papiliotrema baii*	PYCC 6523^T^	Leaf of apple (*Malus domestica*)	China	LK023827	LK023766	LK024012	–	LK023951
*Papiliotrema bandonii*	CBS 9107^T^	Inflorescences of the elephant grass (*Cortaderia selloana*)	Portugal	NR121465	NG042386	KF036518	KF036932	KF037194
*Papiliotrema castaneae*	CGMCC 2.6898^T^	Leaf of Chinese chestnut (*Castanea mollissima*)	China	OP470272	OP470176	OP784851	OP771457	OP853490
*Papiliotrema catalpae*	CGMCC 2.6897^T^	Leaf of yellow catalpa (*Catalpa ovata*)	China	OP470271	OP470175	OP784850	OP771456	OP853507
*Papiliotrema chiangmaiensis*	SDBR-CMU594^T^	Flowers of zamioculcas (*Zamioculcas zamiifolia*)	Thailand	PV834667	PV834497	PV941857	PV947469	PV844824
*Papiliotrema chiangmaiensis*	SDBR-CMU627	Flowers of teak (*Tectona grandis*)	Thailand	PV834668	PV834498	–	–	–
*Papiliotrema chongmingensis*	CGMCC 2.10012^T^	Tidal flat sediment	China	OR588010	OR588010	OR808045	–	OR808032
*Papiliotrema flavescens*	CBS 942^T^	The atmosphere	Japan	NR130696	AB035042	–	–	–
*Papiliotrema flavescens*	SDBR-CMU668	Flowers of golden shrimp (*Pachystachys lutea*)	Thailand	PV834669	PV834499	–	–	–
***Papiliotrema floralis***	**SDBR-CMU888^T^**	**Flowers of Spanish needle (*Bidens pilosa*)**	**Thailand**	**PZ298078**	**PZ298097**	**PZ309737**	**PZ309743**	**PZ309749**
***Papiliotrema floralis***	**SDBR-CMU892**	**Flowers of oncidium (*Oncidium* sp.)**	**Thailand**	**PZ298079**	**PZ298098**	**PZ309738**	**PZ309744**	**PZ309750**
*Papiliotrema fonsecae*	CBS 12692^T^	Subglacial ice with gypsum inclusions	Norway	NR119972	JN193447	–	–	–
*Papiliotrema frias*	CBS 12693^T^	Meltwaters of frias river	Argentina	GU997162	LK023834	–	–	–
*Papiliotrema fusca*	CBS 9648^T^	Leaves of montpellier maple (*Acer monspessulanum*)	Portugal	AF444668	AF444762	–	–	–
*Papiliotrema fusca*	BRIP 76370a	The phylloplane of bloodberry (*Rivina humilis*)	Australia	NR199259	NG244360	–	–	–
*Papiliotrema hoabinhensis*	JCM 10835^T^	Leaf of *Anadendrum montanum*	Vietnam	AB110695	AB193347	OR808045	–	OR808032
*Papiliotrema horticola*	CBS 16715^T^	The hypanthium of apples (*Malus domestica*)	Russia	NR182874	MW579431	LR814015	HG993247	LR814014
*Papiliotrema japonica*	CBS 2013^T^	Japanese zelkova (*Zelkova* sp.)	Japan	NR155613	NG057690	–	–	–
*Papiliotrema laurentii*	CBS 139^T^	Palm wine sap	Congo	NR130670	NG056281	LK024018	KF036793	LK023957
*Papiliotrema leoncinii*	CBS 13918^T^	Freshwater of Dom Helve’cio lake	Brazil	KP203864	KJ608554	–	–	–
*Papiliotrema mangalensis*	CBS 10870^T^	The mangrove habitat	USA	NR144816	NG057803	–	–	–
*Papiliotrema miconiae*	CBS 8358^T^	Flowers of miconia (*Miconia* sp.)	Brazil	AF444387	AF444698	–	–	–
*Papiliotrema millettiae*	NYNU 243102^T^	The phylloplane of trifloiate jewel vine (*Millettia pachycarpa*)	China	PP837692	PP837690	–	–	–
*Papiliotrema millettiae*	NYNU 24472	The phylloplane of dwarf banana (*Musa nana*)	China	PV823290	PV823291	–	–	–
*Papiliotrema mussaendae*	NYNU 23248^T^	The phylloplane of splash of white (*Mussaenda pubescens*)	China	OQ851892	OQ851890	–	–	–
*Papiliotrema mussaendae*	NYNU 23229	The phylloplane of splash of white (*Mussaenda pubescens*)	China	PV823295	PV823292	–	–	–
*Papiliotrema mussaendae*	NYNU 232142	The phylloplane of splash of white (*Mussaenda pubescens*)	China	PV823294	PV823293	–	–	–
*Papiliotrema nemorosa*	CBS 9606^T^	Herbaceous plants	Russia	NR137534	NG058365	KF036389	KF036802	KF037074
*Papiliotrema odontotermitis*	CBS 14181^T^	The gut of the higher termite (*Odontotermes obesus*)	India	KU883277	NR156605	–	–	–
*Papiliotrema perniciosa*	CBS 9605^T^	Turf of a steppe plot	Russia	NR137653	NG060063	KF036393	KF036807	KF037078
*Papiliotrema phichitensis*	DMKU-SP105^T^	The external surface of sugarcane (*Saccharum officinarum*) leaf	Thailand	AB915388	AB826437	–	–	–
*Papiliotrema plantarum*	CBS 15220^T^	Leaves of corn (*Zea mays*)	Thailand	NR164566	LC370335	–	–	–
*Papiliotrema pollinicola*	SDBR-CMU664^T^	Flowers of vinca (*Catharanthus roseus*)	Thailand	PV834671	PV834501	PX582297	PV947470	PV844825
*Papiliotrema pollinicola*	SDBR-CMU595	Flowers of zamioculcas (*Zamioculcas zamiifolia*)	Thailand	PV834670	PV834500	–	–	–
*Papiliotrema pseudoalba*	CBS 7227^T^	Dead leaf of rice (*Oryza sativa*)	Japan	NR073231	NG058276	KF036330	KF036742	KF037013
*Papiliotrema rajasthanensis*	CBS 10406^T^	Inflorescences of plants	India	NR155678	NG058366	KF036398	KF036812	KF037083
*Papiliotrema ruineniae*	CBS 4926^T^	Leaves of *Jacquinia aurantiaca*	Indonesia	LK023826	LK023764	LK024010	–	LK023949
*Papiliotrema siamensis*	CBS 13330^T^	The external surface of sugarcane (*Saccharum officinarum*) leaf	Thailand	NR155608	NG060062	–	–	–
*Papiliotrema taeanensis*	CBS 9742^T^	The saltern	Korea	NR155679	AY422719	KF036406	KF036820	KF037091
*Papiliotrema tapputiae*	BRIP 75038a^T^	Leaf spot on *Decalobanthus peltatus*	Australia	NR187102	NG229127	–	–	–
*Papiliotrema tectonae*	SDBR-CMU632^T^	Flowers of teak (*Tectona grandis*)	Thailand	PV834673	PV834503	PV941859	PV947472	PV844827
*Papiliotrema tectonae*	SDBR-CMU630	Flowers of teak (*Tectona grandis*)	Thailand	PV834672	PV834502	PV941858	PV947471	PV844826
*Papiliotrema terrestris*	CBS 10810^T^	Soil	USA	KY108747	KY104476	–	–	–
*Papiliotrema wisconsinensis*	CBS 13895^T^	Bark of American beech (*Fagus grandifolia*)	USA	NR160324	NG060134	–	–	–
*Papiliotrema zeae*	DSM 104035^T^	Surface of maize kernels (*Zea mays*)	USA	NR168772	MH718306	–	–	–
*Rhynchogastrema aquatica*	CBS 12527^T^	Freshwater	Brazil	NR120001	NG042603	–	–	–
*Rhynchogastrema complexa*	CBS 11570^T^	Air from timber factory	Brazil	NR111476	NG042525	KF036317	KF036730	KF037002
*Rhynchogastrema coronata*	DSM 28188^T^	Agricultural soil	Germany	LN870267	LN870267	–	–	–
*Rhynchogastrema fermentans*	CBS 12399^T^	Fruiting body of an unidentified mushroom	China	NR155732	NG058388	–	–	–
*Rhynchogastrema glucofermentans*	CBS 10381^T^	The gut of *Amphix laevigatus*	Panama	NR119978	NG042404	KF036315	KF036728	KF037000
*Rhynchogastrema nanyangensis*	CBS 12474^T^	The gut of wood-boring larvae	China	NR166792	JN564592	–	–	–
*Rhynchogastrema noutii*	CBS 8364^T^	The exudate of *Eriobotrya japonica*	Brazil	NR111072	NG042389	KF036316	KF036729	KF037001
*Rhynchogastrema tunnelae*	CBS 8024^T^	Human nail	Finland	NR111074	NG042390	–	–	–
*Rhynchogastrema tunnelae*	CBS 6123	Unknown source	Finland	KY104936	KY109187	KF036318	KF036731	KF037003
*Kwoniella mangrovensis*	CBS 8507^T^	The tidal creek of Mangrove Cay	Bahamas	AF444646	AF444742	FJ534928	FJ534943	FJ534866

Note. Sequences obtained in this study are indicated in bold and superscript “T” = the ex-type strains.

### Phylogenetic analyses

The sequences generated in this study were analyzed with additional related sequences retrieved from the GenBank database based on BLASTn searches (http://blast.ncbi.nlm.nih.gov; accessed 10 January 2026). Sequences closely related to the taxa studied were retrieved from GenBank based on BLAST similarity and recent publications, as presented in Table 1. Sequences were aligned separately using MAFFT v. 7 (http://mafft.cbrc.jp/alignment/server; accessed 10 January 2026) (Katoh et al. 2019), and the alignments were inspected and edited using BioEdit v. 7.0.9.1 (Hall 1999). Sequence data for the ITS region and D1/D2 domains were analyzed individually to examine topological incongruence among the phylogenetic trees. Concatenated ITS, D1/D2, *rpb1*, *rpb2*, and *tef1-α* sequence data were then used to generate phylogenetic trees based on maximum likelihood (ML) and Bayesian inference (BI) analyses. The ML analyses were conducted via the CIPRES Science Gateway platform (Miller et al. 2010) using RAxML-HPC v. 8 on ACCESS (v. 8.2.12) (Stamatakis 2014) under the GTRGAMMA model with 1,000 bootstrap replicates. The GTR+I+G substitution model was identified as the best-fit model for the sequence evolution of ITS, D1/D2, *rpb1*, *rpb2*, and *tef1-α* using MrModeltest v. 2.4 according to the Akaike information criterion (Nylander 2004). BI analyses were performed with MrBayes v. 3.2.6 (Ronquist et al. 2012) to estimate Bayesian posterior probabilities (BYPPs). Four simultaneous Markov chains were run for 1 million generations, and trees were sampled every 100^th^ generation. The first 2,500 trees, representing the burn-in phase, were discarded. The phylogenetic trees, showing bootstrap support (BS) values ≥ 50% and BYPPs ≥ 0.90, were visualized using FigTree v. 1.4.3 (Rambaut 2016), and final graphical editing was performed using Adobe Illustrator CC 2019 (version 23.0.3.585) and Adobe Photoshop CS6 (version 13.0) (Adobe Systems, USA).

## Results

### Phylogenetic analyses

**Figure 1.**
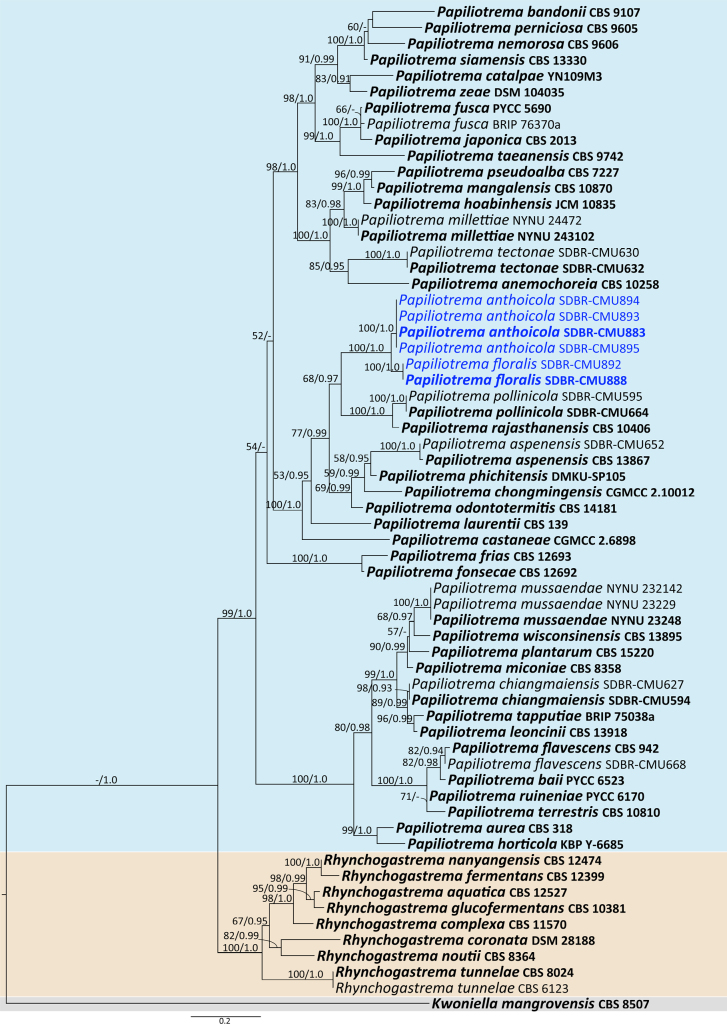
Phylogenetic tree derived from the maximum likelihood analysis of the combined ITS, D1/D2, *rpb1*, *rpb2*, and *tef1-α* sequences of *Rhynchogastremataceae*. *Kwoniella mangrovensis* CBS 8507 was used as the outgroup. Bootstrap values ≥ 50% (left) and Bayesian posterior probabilities ≥ 0.90 (right) are displayed above the nodes. Dashes (-) represent support values lower than 50% ML/0.95 PP. The scale bar represents the expected number of nucleotide substitutions per site. The sequences of *Papiliotrema* species obtained in this study are shown in blue, and the ex-type strains are shown in bold.

Six yeast strains (SDBR-CMU883, SDBR-CMU888, SDBR-CMU892, SDBR-CMU893, SDBR-CMU894, and SDBR-CMU895) belonging to the genus *Papiliotrema* showed distinct characteristics compared with the available data and were selected for accurate identification in this study. To determine their phylogenetic placements, multilocus phylogenetic analyses were performed using the concatenated ITS, D1/D2, *rpb1*, *rpb2*, and *tef1-α* sequence dataset. The dataset consisted of 63 representative strains of species in the family *Rhynchogastremataceae* and an outgroup (*Kwoniella mangrovensis* CBS 8507). The total alignment length comprised 4,914 characters (ITS: 1–658; D1/D2: 659–1,277; *rpb1*: 1,278–2,090; *rpb2*: 2,091–3,167; *tef1-α*: 3,167–3,413). The best-scoring RAxML tree, with a final likelihood optimization value of –39,169.027524, is presented. The matrix contained 2,433 distinct alignment patterns, with 56.66% of the characters being undetermined or gaps. The estimated base frequencies were as follows: A = 0.254219, C = 0.246700, G = 0.263881, and T = 0.235200; the substitution rates were AC = 1.466796, AG = 3.274324, AT = 1.839372, CG = 1.272762, CT = 6.614958, and GT = 1.000000; and the gamma distribution shape parameter was α = 0.515662. The average standard deviation of split frequencies was 0.002684 at the end of the MCMC analysis. The topologies of the phylogenetic trees obtained from the ML and BI analyses were similar. Therefore, the phylogenetic tree generated from the ML analysis is shown in Fig. 1.

Phylogenetic analyses revealed that the six yeast strains obtained in this study were separated into two distinct lineages with high support values (100% MLBS and 1.0 BYPP) within the genus *Papiliotrema* (Fig. 1). Four strains (SDBR-CMU883, SDBR-CMU893, SDBR-CMU894, and SDBR-CMU895) formed one cluster representing the species described here as *P. anthoicola*, whereas the remaining two strains (SDBR-CMU888 and SDBR-CMU892) formed a separate cluster representing the species described here as *P. floralis*. *Papiliotrema anthoicola* and *P. floralis* differed by 1.19% nucleotide divergence (six nucleotide substitutions) in the ITS region and 0% nucleotide divergence (zero nucleotide substitutions) in the D1/D2 domains, as well as by substantial divergence in the *rpb1*, *rpb2*, and *tef1-α* genes, with 2.85%, 5.34%, and 5.90% nucleotide divergence (23, 56, and 71 nucleotide substitutions), respectively (Table 2). Although the nucleotide divergence in the ITS region and D1/D2 domains between *P. anthoicola* and *P. floralis* (1.19% in ITS and 0% in D1/D2) was lower than the species delimitation thresholds proposed for basidiomycetous yeasts by Vu et al. (2016) (1.59% nucleotide divergence for ITS and 0.49% nucleotide divergence for D1/D2), the levels of sequence divergence observed in the *rpb1*, *rpb2*, and *tef1-α* genes further support the recognition of these taxa as distinct species, as currently applied to closely related *Papiliotrema* species (Jiang et al. 2024; Kodchasee et al. 2026). Additionally, their morphological and physiological differences are described below.

**Table 2.** Nucleotide substitutions and sequence divergence of *P. anthoicola* and *P. floralis* compared with closely related *Papiliotrema* species.

**Loci**	**Strain**	**Sequence divergence (nucleotide substitutions)**			
		**SDBR-CMU883**	**SDBR-CMU888**	**SDBR-CMU664**	**CBS 10406**
D1/D2	SDBR-CMU883	0.00% (0 nt)	–	–	–
	SDBR-CMU888	0.00% (0 nt)	0.00% (0 nt)	–	–
	SDBR-CMU664	2.96% (18 nt)	2.91% (18 nt)	0.00% (0 nt)	–
	CBS 10406	1.65% (10 nt)	1.65% (10 nt)	3.70% (38 nt)	0.00% (0 nt)
ITS	SDBR-CMU883	0.00% (0 nt)	–	–	–
	SDBR-CMU888	1.19% (6 nt)	0.00% (0 nt)	–	–
	SDBR-CMU664	5.05% (26 nt)	4.94% (25 nt)	0.00% (0 nt)	–
	CBS 10406	5.44% (28 nt)	5.34% (26 nt)	0.56% (3 nt)	0.00% (0 nt)
*rpb1*	SDBR-CMU883	0.00% (0 nt)	–	–	–
	SDBR-CMU888	2.85% (23 nt)	0.00% (0 nt)	–	–
	SDBR-CMU664	23.20% (187 nt)	23.27% (188 nt)	0.00% (0 nt)	–
	CBS 10406	23.05% (180 nt)	23.37% (183 nt)	0.39% (3 nt)	0.00% (0 nt)
*rpb2*	SDBR-CMU883	0.00% (0 nt)	–	–	–
	SDBR-CMU888	5.34% (56 nt)	0.00% (0 nt)	–	–
	SDBR-CMU664	18.24% (187 nt)	18.15% (190 nt)	0.00% (0 nt)	–
	CBS 10406	18.57% (195 nt)	18.62% (199 nt)	3.70% (38 nt)	0.00% (0 nt)
*tef1-α*	SDBR-CMU883	0.00% (0 nt)	–	–	–
	SDBR-CMU888	5.90% (71 nt)	0.00% (0 nt)	–	–
	SDBR-CMU664	18.54% (218 nt)	18.62% (218 nt)	0.00% (0 nt)	–
	CBS 10406	10.11% (48 nt)	10.17% (47 nt)	0.00% (1 nt)	0.00% (0 nt)

Note. SDBR-CMU883 = *P. anthoicola*; SDBR-CMU888 = *P. floralis*; SDBR-CMU664 **=***P. pollinicola*; CBS 10406 = *P. rajasthanensis*.

In the phylogenetic tree, *P. anthoicola* and *P. floralis* formed a sister clade and were closely related to *P. pollinicola* and *P. rajasthanensis*, with support values of 68% MLBS and 0.97 BYPP (Fig. 1). *Papiliotrema anthoicola* differed from *P. pollinicola* and *P. rajasthanensis* by 26 (5.05% nucleotide divergence) and 28 (5.44% nucleotide divergence) nucleotide substitutions in the ITS region, respectively. In the D1/D2 domains, the sequences showed 18 (2.96% nucleotide divergence) and 10 (1.65% nucleotide divergence) nucleotide substitutions compared with *P. pollinicola* and *P. rajasthanensis*, respectively. In comparison, *P. floralis* differed from *P. pollinicola* and *P. rajasthanensis* by 25 nucleotide substitutions (4.94% nucleotide divergence) and 26 nucleotide substitutions (5.34% nucleotide divergence) in the ITS region, respectively. The D1/D2 domains of *P. floralis* showed 2.91% and 1.65% nucleotide divergence, corresponding to 18 and 10 nucleotide substitutions, compared with *P. pollinicola* and *P. rajasthanensis*, respectively. Therefore, *P. anthoicola* and *P. floralis* were considered distinct from *P. pollinicola* and *P. rajasthanensis* because their sequence divergence values exceeded the species delimitation thresholds proposed for basidiomycetous yeasts (Vu et al. 2016).

### Taxonomic descriptions

#### Papiliotrema anthoicola

Taxon classificationFungiTremellalesRhynchogastremaceae

Kathongthung, Kodchasee, Senwanna, J. Kumla & N. Suwannar. sp. nov.

13AB04C5-D1D9-5781-A4CA-F3986118CB47

862791

Fig. 2

##### Etymology.

The specific epithet “anthoicola” named for inhabiting flowers, the substrate from which type of strain was first isolated.

**Figure 2.**
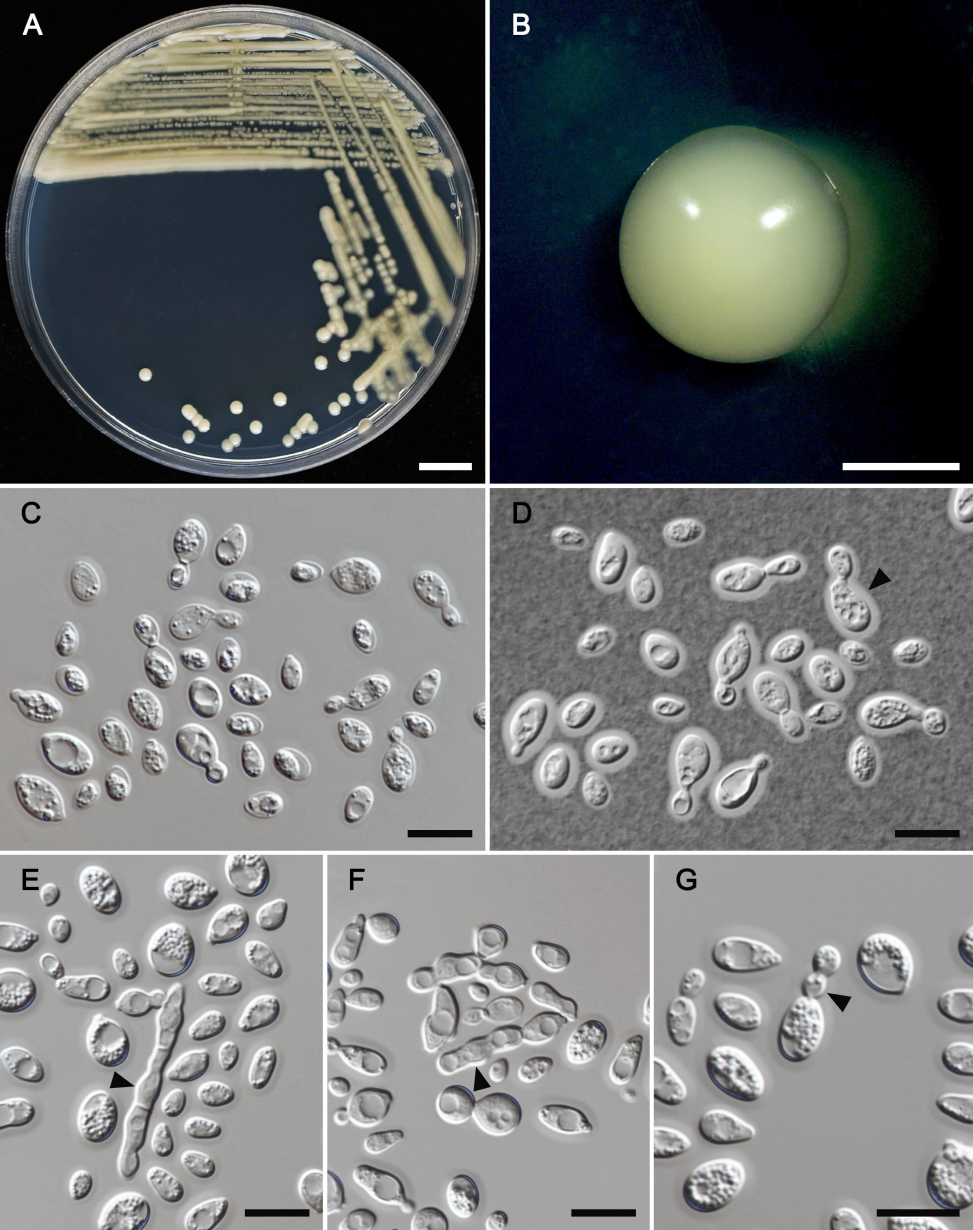
Morphology of *Papiliotrema anthoicola* (SDBR-CMU883, ex-type). **A**. Culture; **B**. Single colony; **C**. Cells and budding cells; **D**. Capsules, negatively stained with India ink and indicated by an arrowhead, on YMA after incubation at 25 °C for 5 days; **E, F**. Cells and elongated cells, indicated by arrowheads; **G**. Chain of cells, indicated by an arrowhead, in Dalmau plate cultures on PDA after incubation at 25 °C for 2 weeks. Scale bars: 10 mm (**A**); 1 mm (**B**); 10 μm (**C–G**).

##### Holotype.

Thailand • Chiang Mai Province, Phrao District, Nam Phrae, in the ylang-ylang flower (*Cananga odorata*), August 2024, C. Kathongthung, P. Kodchasee, C. Senwanna, J. Kumla, and N. Suwannarach, CMUB40150, holotype (preserved in metabolically inactive state). Ex-type living culture SDBR-CMU883 = GMBCC2525 = TBRC 21859. GenBank accession numbers: PZ298074 (ITS), PZ298093 (D1/D2), PZ309733 (*rpb1*), PZ309739 (*rpb2*), PZ309745 (*tef1-α*).

##### Description.

The culture on YMA after 5 days at 25 °C, colonies are circular form (2.0–3.0 mm in diameter), yellowish white, smooth surface, glistening appearance, convex elevation, and entire margin. The cells are ovoid and ellipsoidal (2.63–5.78 × 4.48–7.79 μm, *n* = 50), occur singly or in pairs. Budding is polar. Extracellular capsule is produced (0.47–1.63 μm in thickness, *n* = 50). Ballistoconidia are not produced. In Dalmau plates after 2 weeks on PDA and cornmeal agar at 25 °C, elongated cells and chains of cells are presence on PDA, but neither pseudohyphae nor true hyphae are formed. Basidiospores are not obtained for individual strains on YMA, YPDA, CMA, 5% MEA, PDA, Fowell’s acetate agar, and Gorodkowa agar after incubation at 25 °C for 1 month.

Fermentation of glucose is negative. *D*-glucose, *D*-galactose, *N*-acetylglucosamine, *D*-ribose (weak), *D*-xylose (weak), *L*-arabinose, *L*-rhamnose, sucrose, maltose, α,α-trehalose, methyl-α-*D*-glucoside (weak), cellobiose, salicin (weak), melibiose, lactose, raffinose, melezitose, glycerol (weak), erythritol, *D*-mannitol, *myo*-inositol, *D*-glucono-1,5-lactone, *D*-gluconate, *D*-galacturonic acid, succinate, and xylitol (weak) are assimilated as carbon sources, whereas *L*-sorbose, *D*-arabinose, inulin, soluble starch, ribitol, *D*-glucitol, galactitol, *D*-glucuronate, *DL*-lactate, citrate, methanol, and ethanol are not assimilated. Ammonium sulfate, ethylamine hydrochloride, and *L*-lysine are assimilated as sole nitrogen sources, whereas potassium nitrate, sodium nitrite, and cadaverine are not assimilated. Growth occurs on media containing 50% glucose (weak) or 60% glucose (weak) and on medium containing 10% NaCl supplemented with 5% glucose. No growth occurs on vitamin-free medium or on media containing 16% NaCl supplemented with 5% glucose, 0.01% cycloheximide, or 0.1% cycloheximide. Acid formation is positive. Growth occurs at 10 °C, 15 °C, 25 °C, 30 °C, and 37 °C but not at 45 °C.

##### Additional strains examined.

Thailand • Chiang Mai Province, Mueang District, Chang Phueak, in the frangipani flower (*Plumeria obtusa*), August 2024, C. Kathongthung, P. Kodchasee, C. Senwanna, J. Kumla, and N. Suwannarach, living culture SDBR-CMU893, • *ibid*., in the golden trumpet flower (*Allamanda cathartica*), July 2024, C. Kathongthung, P. Kodchasee, C. Senwanna, J. Kumla, and N. Suwannarach, living culture SDBR-CMU895; Phrao District, Nam Phrae, in the ylang-ylang flower (*Cananga odorata*), August 2024, C. Kathongthung, P. Kodchasee, C. Senwanna, J. Kumla, and N. Suwannarach, living culture SDBR-CMU894. GenBank accession numbers SDBR-CMU893: PZ298075 (ITS), PZ298094 (D1/D2), PZ309734 (*rpb1*), PZ309740 (*rpb2*), PZ309746 (*tef1-α*); SDBR-CMU894: PZ298076 (ITS), PZ298095 (D1/D2), PZ309735 (*rpb1*), PZ309741 (*rpb2*), PZ309747 (*tef1-α*); SDBR-CMU895: PZ298077 (ITS), PZ298096 (D1/D2), PZ309736 (*rpb1*), PZ309742 (*rpb2*), PZ309748 (*tef1-α*).

##### Note.

In the phylogenetic tree, strains SDBR-CMU883, SDBR-CMU893, SDBR-CMU894, and SDBR-CMU895 of *P. anthoicola* formed a distinct lineage that was sister to *P. floralis* and clustered with *P. rajasthanensis* and *P. pollinicola* (Fig. 1). Data on nucleotide divergence in the ITS, D1/D2, *rpb1*, *rpb2*, and *tef1-α* sequences are presented above (Table 2). Morphologically, *P. anthoicola* is similar to *P. floralis* and *P. rajasthanensis* in possessing extracellular capsules surrounding the cells, whereas such capsules were not observed in *P. pollinicola* (Saluja and Prasad 2007; Kodchasee et al. 2026). The ovoid to ellipsoidal cells of *P. anthoicola* can be distinguished from the oval cells of *P. rajasthanensis* (Saluja and Prasad 2007). The development of elongated cells in *P. anthoicola* on PDA after 2 weeks at 25 °C differed from that in *P. floralis*, which did not form elongated cells. Physiologically, *P. anthoicola* differs from *P. floralis* in its ability to assimilate *D*-ribose, *D*-glucono-1,5-lactone, *D*-galacturonic acid, and succinate, whereas *P. floralis* cannot utilize these substrates (Table 3). Moreover, *P. anthoicola* can grow at 37 °C, whereas *P. floralis* cannot. Compared with *P. pollinicola*, *P. anthoicola* lacks the ability to assimilate *L*-sorbose, *D*-arabinose, ribitol, *D*-glucitol, galactitol, *D*-glucuronate, *DL*-lactate, citrate, and ethanol, which *P. pollinicola* can utilize (Kodchasee et al. 2026). Additionally, *P. anthoicola* can be distinguished from *P. rajasthanensis* by its ability to assimilate glycerol and grow on media containing 50% and 60% glucose and at 37 °C, whereas *P. rajasthanensis* cannot grow under these conditions (Saluja and Prasad 2007). Consequently, *P. anthoicola*, represented by strains SDBR-CMU883, SDBR-CMU893, SDBR-CMU894, and SDBR-CMU895, is introduced as a novel species based on a polyphasic approach.

**Table 3.** Biochemical and physiological characteristics differentiating *P. anthoicola* and *P. floralis* from closely related *Papiliotrema* species.

**Characteristics**	***P. anthoicola* SDBR-CMU883**	***P. floralis* SDBR-CMU888**	*P. pollinicola* SDBR-CMU664*	*P. rajasthanensis* CBS 10406*
**Assimilation**				
*L*-Sorbose	–	–	+	w
*D*-Ribose	w	–	+	+
*D*-Xylose	w	+	+	+
*D*-Arabinose	–	–	+	+
α-α-Trehalose	+	w	+	+
Methyl-α-*D*-Glucoside	w	w	+	+
Salicin	w	w	+	+
Inulin	–	–	–	w
Glycerol	w	w	+	–
Erythritol	+	w	+	w
Ribitol	–	+	+	+
*D*-Glucitol	–	+	+	+
Galactitol	–	+	+	+
*D*-Glucono-1,5-lactone	+	–	+	+
*D*-Glucuronate	–	–	+	+
*D*-Galacturonic acid	+	–	+	nd
*DL*-Lactate	–	–	+	–
Succinate	+	–	+	w
Citrate	–	–	+	w
Ethanol	–	–	+	w
Xylitol	w	w	+	+
Cadaverine	–	–	–	+
**Growth characteristics**				
Growth on 50% glucose media	+	+	+	–
Growth on 60% glucose media	w	+	+	–
Growth on 10% NaCl/5% glucose media	+	+	w	nd
Growth at 10 °C	+	s	+	nd
Growth at 37 °C	w	–	–	–
Acid formation	+	w	–	–

Note. +, positive reaction; –, negative reaction; l, delayed positive (latent) reaction; s, slow positive reaction; v, variable reaction; w, weakly positive reaction; nd, not determined. All data were obtained in this study, except those marked with an asterisk (*), which were obtained from the original descriptions (Saluja and Prasad 2007; Kodchasee et al. 2026).

#### Papiliotrema floralis

Taxon classificationFungiTremellalesRhynchogastremaceae

Kodchasee, Kathongthung, Senwanna, J. Kumla & N. Suwannar. sp. nov.

6DE4AD7A-EACE-5E23-AC68-360658C1A173

862792

Fig. 3

##### Etymology.

The specific epithet “floralis” refers to being associated with flowers, from which this species was isolated.

**Figure 3.**
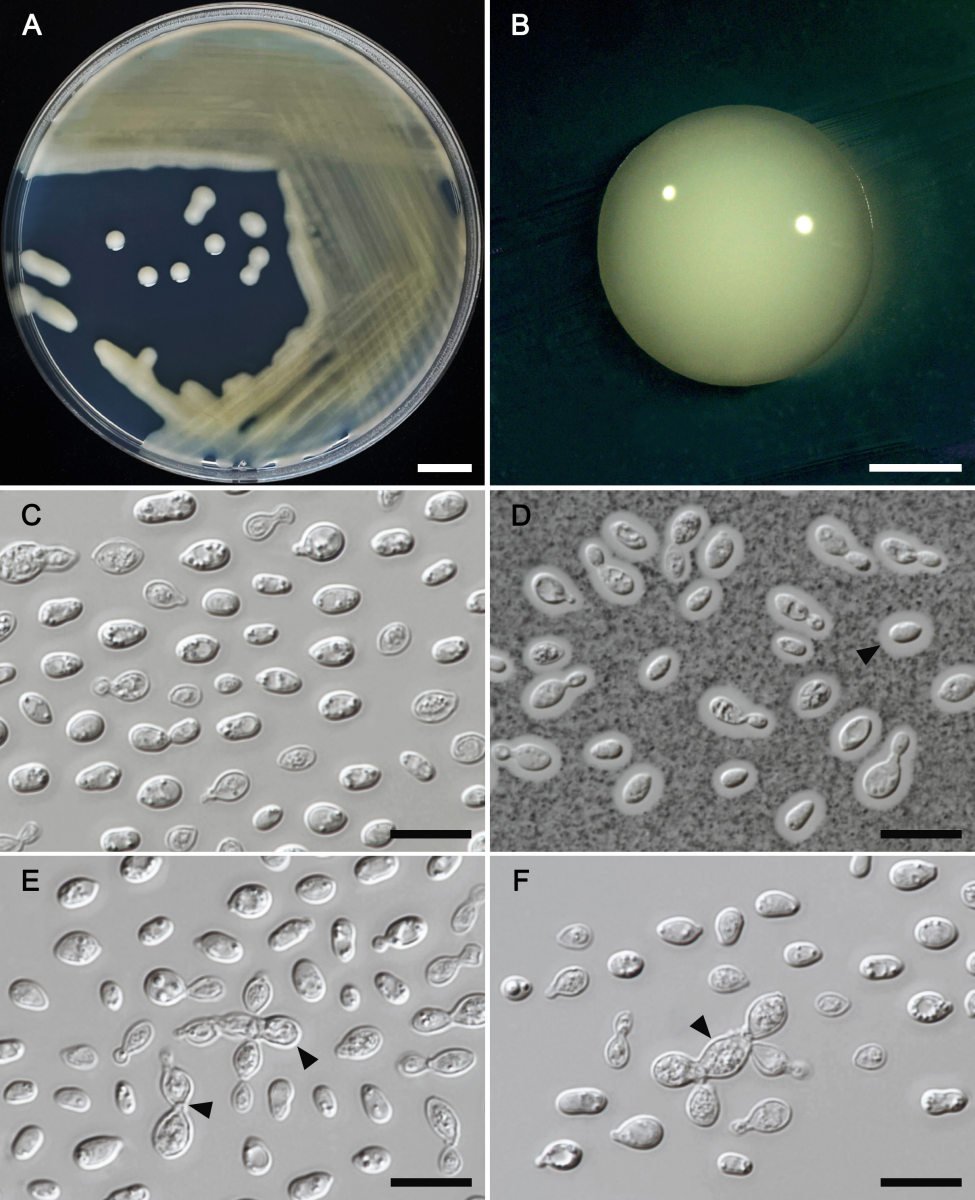
Morphology of *Papiliotrema floralis* (SDBR-CMU888, ex-type). **A**. Culture; **B**. Single colony; **C**. Cells and budding cells; **D**. Capsules, negatively stained with India ink and indicated by an arrowhead; **E, F**. Cells and chains of cells, indicated by arrowheads, on YMA after incubation at 25 °C for 5 days. Scale bars: 10 mm (**A**); 1 mm (**B**); 10 μm (**C–F**).

##### Holotype.

Thailand • Chiang Mai Province, Mueang District, Chang Phueak, in the Spanish needle flower (*Bidens pilosa*), July 2024, C. Kathongthung, P. Kodchasee, C. Senwanna, J. Kumla, and N. Suwannarach, CMUB40151, holotype (preserved in metabolically inactive state). Ex-type living culture SDBR-CMU888 = GMBCC2530 = TBRC 21861. GenBank accession numbers: PZ298078 (ITS), PZ298097 (D1/D2), PZ309737 (*rpb1*), PZ309743 (*rpb2*), PZ309749 (*tef1-α*).

##### Description.

The culture on YMA after 5 days at 25 °C, colonies are circular form (3.0–4.0 mm in diameter), yellowish white, smooth surface, glistening and viscous appearance, convex elevation, and entire margin. The cells are ovoid and ellipsoidal (2.40–4.31 × 3.54–6.91 μm, *n* = 50), occur singly, in pairs, or in short chain. Budding is multilateral. Extracellular capsule is produced (0.53–1.93 μm in thickness, *n* = 50). Ballistoconidia are not produced. In Dalmau plates after 2 weeks on PDA and CMA at 25 °C, neither pseudohyphae nor true hyphae are formed. Basidiospores are not obtained for individual strains on YMA, YPDA, CMA, 5% MEA, PDA, Fowell’s acetate agar, and Gorodkowa agar after incubation at 25 °C for 1 month.

Fermentation of glucose is negative. *D*-glucose, *D*-galactose, *N*-acetylglucosamine, *D*-xylose (weak), *L*-arabinose, *L*-rhamnose, sucrose, maltose, α,α-trehalose (weak), methyl-α-*D*-glucoside (weak), cellobiose, salicin (weak), melibiose, lactose, raffinose, melezitose, glycerol (weak), erythritol (weak), *D*-glucitol, *D*-mannitol, galactitol, *myo*-inositol, *D*-gluconate, and xylitol (weak) are assimilated as carbon sources, whereas *L*-sorbose, *D*-ribose, *D*-arabinose, inulin, soluble starch, ribitol, *D*-glucono-1,5-lactone, *D*-glucuronate, *D*-galacturonic acid, *DL*-lactate, succinate, citrate, methanol, and ethanol are not assimilated. Ammonium sulfate, ethylamine hydrochloride, and *L*-lysine are assimilated as sole nitrogen sources, whereas potassium nitrate, sodium nitrite, and cadaverine are not assimilated. Growth occurs on media containing 50% or 60% glucose and on medium containing 10% NaCl supplemented with 5% glucose. No growth occurs on vitamin-free medium or on media containing 16% NaCl supplemented with 5% glucose, 0.01% cycloheximide, or 0.1% cycloheximide. Acid formation is weakly positive. Growth occurs at 10 °C (slow), 15 °C, 25 °C, and 30 °C but not at 37 °C or 45 °C.

##### Additional strain examined.

Thailand • Chiang Mai Province, Phrao District, Nam Phrae, in the oncidium flower (*Oncidium* sp.), July 2024, C. Kathongthung, P. Kodchasee, C. Senwanna, J. Kumla, and N. Suwannarach, living culture SDBR-CMU892. GenBank accession numbers: PZ298079 (ITS), PZ298098 (D1/D2), PZ309738 (*rpb1*), PZ309744 (*rpb2*), PZ309750 (*tef1-α*).

##### Note.

Two strains, SDBR-CMU888 and SDBR-CMU892, representing *P. floralis*, formed a distinct lineage that was sister to *P. anthoicola* and clustered with *P. rajasthanensis* and *P. pollinicola* (Fig. 1). Data on nucleotide divergence in the ITS, D1/D2, *rpb1*, *rpb2*, and *tef1-α* sequences are presented above (Table 2). The morphological, biochemical, and physiological characteristics distinguishing *P. floralis* from *P. anthoicola* are presented above. *Papiliotrema floralis* can be distinguished from *P. pollinicola* by its production of capsules, whereas *P. pollinicola* does not produce capsules (Kodchasee et al. 2026). The multilateral budding in *P. floralis* differs from the polar budding observed in *P. rajasthanensis* (Saluja and Prasad 2007). *Papiliotrema floralis* can be distinguished from *P. pollinicola* by its inability to assimilate *L*-sorbose, *D*-ribose, *D*-arabinose, *D*-glucono-1,5-lactone, *D*-glucuronate, *D*-galacturonic acid, *DL*-lactate, succinate, citrate, and ethanol, whereas *P. pollinicola* can assimilate these substrates (Kodchasee et al. 2026). Additionally, *P. floralis* can be distinguished from *P. rajasthanensis* by its inability to assimilate *L*-sorbose, *D*-ribose, *D*-arabinose, inulin, *D*-glucono-1,5-lactone, *D*-glucuronate, succinate, citrate, ethanol, and cadaverine (Saluja and Prasad 2007). Based on a polyphasic taxonomic approach, *P. floralis*, represented by strains SDBR-CMU888 and SDBR-CMU892, is described as a new species.

## Discussion

The current classification of yeasts within the genus *Papiliotrema* relies on phylogenetic analyses combined with morphological, biochemical, and physiological data (Jiang et al. 2024; Tan and Shivas 2024; Gao et al. 2025; Sun et al. 2025; Kodchasee et al. 2026). In this study, two novel *Papiliotrema* species, *P. anthoicola* and *P. floralis*, were isolated from flower samples collected in northern Thailand in 2024 and described based on a polyphasic taxonomic approach using morphological, biochemical, physiological, and multilocus phylogenetic data. Phylogenetically, *P. anthoicola* and *P. floralis* cluster together and form a sister clade to *P. pollinicola* and *P. rajasthanensis* (Fig. 1). Their differences in morphology, biochemistry, physiology, and nucleotide divergence are presented above. The nucleotide divergence in the ITS region and D1/D2 domains between *P. anthoicola* and *P. floralis* was 1.19% and 0%, respectively. According to Vu et al. (2016), the proposed species delimitation thresholds for basidiomycetous yeasts are 1.59% nucleotide divergence for ITS and 0.49% for the D1/D2 domains. However, nucleotide divergence values lower than these thresholds have been reported in some closely related species of basidiomycetous yeasts, including species within *Bannoa*, *Curvibasidium*, *Derxomyces*, *Fellozyma*, *Papiliotrema*, *Piskurozyma*, *Takashimella*, and *Teunia* (Jiang et al. 2024; Feng et al. 2025; Sun et al. 2025; Kodchasee et al. 2026). Therefore, multilocus phylogenetic analyses are essential for accurate species delimitation, particularly among closely related taxa (Li et al. 2020, 2022; Jiang et al. 2024; Schoutteten et al. 2024; Feng et al. 2025; Sun et al. 2025; Kodchasee et al. 2026). In this study, the inclusion of *rpb1*, *rpb2*, and *tef1-α* in the multilocus phylogenetic analyses, together with morphological, biochemical, and physiological characterization, confirmed that *P. anthoicola* and *P. floralis* represent distinct species. Furthermore, whole-genome sequencing and average nucleotide identity (ANI) analysis have been used to distinguish closely related yeast species and resolve species boundaries (Cortimiglia et al. 2024; Jiang et al. 2024; Khammeankea et al. 2026). Future studies incorporating these genomic approaches will provide further support for the taxonomic placement of the yeast strains identified in this study and improve our understanding of their evolutionary relationships.

Flowers are considered important natural habitats for yeast studies because they provide abundant nutrient sources, particularly nectar, which supports yeast growth and diversity (Mittelbach et al. 2015; Bogo et al. 2021; Félix et al. 2021; Jacquemyn et al. 2021; Kanpiengjai et al. 2023). Anthophilous yeasts can influence nectar chemistry, including sugar composition, pH, and volatile organic compounds, which may subsequently affect pollinator behavior and plant-pollinator interactions that are important for pollination processes (Pozo et al. 2011; Mittelbach et al. 2015; Jacquemyn et al. 2021). Research on the diversity of anthophilous yeasts has increased because of their ecological roles in plant-microbe and plant-pollinator interactions (Klaps et al. 2020; Kodchasee et al. 2026). As a result, numerous anthophilous yeast species have been reported from floral habitats worldwide. Previous studies have reported that species of *Papiliotrema* have been isolated from flowers in various regions worldwide (Sampaio et al. 2002; Min et al. 2012, 2013a, 2013b; Hyun et al. 2014a, 2014b; Han et al. 2015a, 2015b; Canto et al. 2017; Kim et al. 2020; Jang et al. 2021; Kodchasee et al. 2026). Furthermore, the dispersal capacity of yeasts in floral environments, influenced by factors such as geographical location, wind, rainfall, microhabitat heterogeneity, and biotic interactions (e.g., pollinators and other flower-visiting animals), may contribute to shaping their distribution and diversification (Canto et al. 2017; Klaps et al. 2020; Boekhout et al. 2022; Kanpiengjai et al. 2023; Kodchasee et al. 2026).

Prior to this study, 16 species of *Papiliotrema* had been reported from diverse habitats in Thailand, including *P. aspenensis*, *P. chiangmaiensis*, *P. flavescens*, *P. japonica*, *P. laurentii*, *P. nemorosa*, *P. phichitensis*, *P. plantarum*, *P. pollinicola*, *P. pseudoalba*, *P. rajasthanensis*, *P. ruineniae*, *P. siamensis*, *P. tectonae*, *P. terrestris*, and *Papiliotrema aff. mangaliensis* (Nakase et al. 2001; Jaiboon et al. 2016; Khunnamwong et al. 2018b; Srisuk et al. 2019; Boonmak et al. 2020; Into et al. 2020; Kumla et al. 2020; Limtong et al. 2020; Satianpakiranakorn et al. 2020; Sapsirisuk et al. 2022; Kanpiengjai et al. 2023; Kodchasee et al. 2026; Tran et al. 2026). Five species, namely *P. chiangmaiensis*, *P. flavescens*, *P. aspenensis*, *P. pollinicola*, and *P. tectonae*, were isolated from flowers (Kanpiengjai et al. 2023; Kodchasee et al. 2026). Therefore, the discovery of two novel species, *P. anthoicola* and *P. floralis*, increases the number of known *Papiliotrema* species in Thailand to 18. The discovery of *P. anthoicola* and *P. floralis* in the present study further expands the known diversity of flower-associated *Papiliotrema* species and highlights flowers in northern Thailand as promising reservoirs of previously undescribed anthophilous yeast taxa. These findings may stimulate further research on the distribution and ecology of anthophilous yeasts in Thailand, across Asia, and worldwide. Furthermore, these novel yeast strains may represent potential biotechnological resources that warrant further investigation. Future studies on yeast diversity in Thailand should include multiple regions and a wider range of flower species.

## Supplementary Material

XML Treatment for Papiliotrema anthoicola

XML Treatment for Papiliotrema floralis

## Additional information

### Artificial Intelligence (AI) use

The authors accept full responsibility for the content of the manuscript, including the disclosure of any use of AI.

No AI tools were used in the preparation of this manuscript.

### Author ORCIDs

C. Kathongthung https://orcid.org/0009-0007-0517-6063

P. Kodchasee https://orcid.org/0000-0003-0432-4910

S. Singsalood https://orcid.org/0009-0005-5726-7042

C. Senwanna https://orcid.org/0000-0002-1008-4514

P. Khunnamwong https://orcid.org/0000-0001-7536-929X

J. Kumla https://orcid.org/0000-0002-3673-6541

N. Suwannarach https://orcid.org/0000-0002-2653-1913

### Data availability

All of the data that support the findings of this study are available in the main text.
